# Effects of a Novel *COL4A3* Homozygous/Heterozygous Splicing Mutation on the Mild Phenotype in a Family With Autosomal Recessive Alport Syndrome and a Literature Review

**DOI:** 10.1002/mgg3.70053

**Published:** 2025-02-09

**Authors:** Dan Chen, Li Zhang, Jing Rao, Yan Zhou, Lujun Dai, Songsong Huang, Chunxia Yang, Qiuhan Bian, Tao Zhang, Xiaoyan Yang

**Affiliations:** ^1^ Department of Pediatrics The Affiliated Hospital of Guizhou Medical University, Guizhou Provincial Children's Medical Center Guiyang Guizhou China; ^2^ Department of Pathology The Affiliated Hospital of Guizhou Medical University Guiyang Guizhou China

**Keywords:** Alport syndrome (AS), COL4A3, Minigene assay, splicing mutation, type IV collagen

## Abstract

**Background:**

Alport syndrome involves chronic progressive kidney failure and extrarenal organ damage caused by *COL4A3*, *COL4A4*, and *COL4A5* mutations.

**Methods:**

We initially discerned a *COL4A3* splicing mutation via next‐generation sequencing. Next, we used bioinformatics, renal biopsy pathology, and an in vitro minigene experiment. Complementary analysis of clinical data was carried out, and we explored the expression and function of the variants to verify their pathogenicity.

**Results:**

A splicing mutation *(c.687 + 1G > T)* in *COL4A3* was found in a Chinese family. Bioinformatics analysis revealed its impact on splicing, causing a translational frameshift, which was confirmed by an in vitro minigene assay. The proband's glomerular basement membrane displayed reduced type IV collagen α3, α4, and α5 chains, with some absent, suggesting disruption of collagen IV trimers in the glomerular basement membrane, potentially damaging the glomerular filtration barrier.

**Conclusion:**

We present a novel finding of a previously unreported *c.687 + 1G > T* mutation in *COL4A3* that disrupts transcription and translation, impairing α3α4α5 (IV) chain formation, altering the integrity of the glomerular basement membrane, causing hereditary Alport syndrome. This discovery enriches the genetic map of Alport syndrome, aiding in clinical genetic guidance, and enhancing the efficacy of prenatal testing.

## Graphical abstract

We present a novel finding of a previously unreported *c.687 + 1G > T* mutation in *COL4A3*, disrupting transcription and translation, impairing α3, α4, α5 (IV) chain formation. This alters the glomerular basement membrane integrity, causing hereditary Alport syndrome. This discovery enriches the genetic map of Alport syndrome, aiding clinical genetic guidance and enhancing pregnancy testing efficacy.

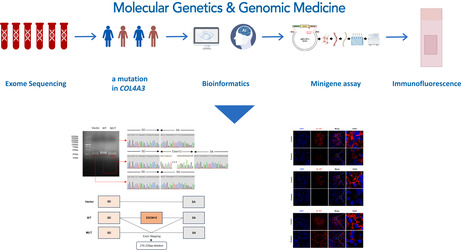

## 1 Background

Alport syndrome (AS; OMIM#301050, #104,200, #620,536, #203,780) is an uncommon nephropathy that affects other organs like the eyes and ears, in addition to causing structural and functional abnormalities in the glomerular basement membranes. Hematuria, proteinuria, and chronic, progressive renal failure are the main symptoms in patients. Sensorineural deafness, retinopathies, and other extrarenal organ involvement frequently follow (Savige et al. 2022; Warady et al. 2020). The most frequent manifestation is microhematuria. However, some individuals can progressively acquire proteinuria. Upon reaching the age of 30, a significant proportion of individuals diagnosed with AS inevitably develop end‐stage kidney disease (ESKD) (Kashtan and Gross 2021). AS, a hereditary glomerular disorder, is attributed to genetic abnormalities within the *COL4A3*, *COL4A4*, and *COL4A5* genes, which are responsible for encoding collagen IV α3, α4, and α5 chains (Kashtan 2021). On the basis of the presence of various mutated genes, the inheritance of AS can be categorized into three distinct patterns: X‐linked AS, which is caused by pathogenic variants in the *COL4A5* gene; and autosomal recessive and dominant AS (ARAS, ADAS), which are caused by homozygous/compound heterozygous and heterozygous pathogenic variants in *COL4A3* and/or *COL4A4, respectively* (Kashtan et al. 2018). Owing to advancements in sequencing technology, a variety of mutations in the *COL4A3* gene have been identified, including missense, splicing, deletion, frame‐shifting, and nonsense mutations. However, no mutation hotspots have been confirmed (Groopman et al. 2019; Storey et al. 2013), suggesting that numerous mutations remain to be discovered. The identification of the pathogenic *COL4A3* mutation enhances the understanding of the role of collagen IV in biological processes and helps with the genetic diagnosis of AS. Understanding the underlying molecular mechanism of AS is crucial.

This study reports a *COL4A3* splicing mutation (*c.687 + 1G > T*) in a Chinese family that has never been previously reported. We then further analyzed the effects of this variant on the transcription of the encoded type IV collagen chain and its heterotrimers through bioinformatics and experiments.

## 2 Methods

### 2.1 Editorial Policies and Ethical Considerations

The study procedure was approved by the Medical Ethics Committee of the Affiliated Hospital of Guizhou Medical University and adhered to the principles outlined in the Helsinki Declaration.

### 2.2 Patients and Genetic Analysis

This study included a proband and a control subject diagnosed with Thin Basement Membrane Nephropathy (TBMN). The control subject's next‐generation sequencing results revealed no mutations in genes associated with type IV collagen, including *COL4A3*, *COL4A4*, and *COL4A5*. The proband was an 8‐year‐old female who was diagnosed with hematuria and proteinuria at the age of 4 years, without renal failure, hypertension, hearing loss, or visual impairment, and renal biopsy revealed thinning of the glomerular basement membrane. Treatment with captopril was effective. Given that the child's parents are first cousins, we performed peripheral blood next‐generation sequencing on 22 family members of the proband (Figure 2A). The results revealed one individual with a homozygous mutation in the *COL4A3* gene *(c.687 + 1G > T)*, who is the proband in this family, and nine individuals with heterozygous mutations at this site. Among them, five heterozygous mutation carriers had microscopic hematuria, with or without proteinuria; one heterozygous mutation carrier had microalbuminuria; and three heterozygous mutation carriers had normal urinalysis results. None of these family members exhibited signs of renal insufficiency, hypertension, hearing loss, or visual impairment.

Whole blood was obtained via EDTA anticoagulation vessels, and DNA was extracted via an extraction kit. Sequencing was carried out according to the manufacturer's method utilizing the GenCap custom enrichment kit (MyGenostics Inc.) and the Illumina HiSeq 2000 sequencer (Illumina). SOAP (Short Oligonucleotide Analysis Package) aligner software (access URL for soap.genomics.org.cn/soapsnp.html) and Genome Analysis Toolkit software 3.7 were used for reprocessing. The screening process is visually represented in Table S1 online. Sanger sequencing was utilized to validate the identified alterations. GRCh38 or hg38 (GenBank accession number: NM_000091.5) was used as the reference sequence.

### 2.3 Bioinformatics Analysis

The Human Splicing Finder (http://www.umd.be/HSF/), SpliceAI (https://spliceailookup.broadinstitute.org/#), MutationTaster (http://www.mutationtaster.org/), varSEAK (https://varseak.bio/index.php) and the RNA Splicer online server (https://rddc.tsinghua‐gd.org/ai/rna‐splicer) were employed to ascertain the pathogenicity of the novel mutation. Additionally, the IBS online tool was utilized to generate a graphical representation of the collagen IV alpha3 chain domains (accessible at http://ibs.biocuckoo.org/online.php). The protein structure of the collagen IV alpha3 chain was predicted in both its wild‐type and mutant forms via the AlphaFold protein structure database, which is accessible at https://alphafold.ebi.ac.uk/.

### 2.4 Cell Culture

We procured conditionally immortalized human HEK293T cells, which were cultivated in a medium consisting of 90% DMEM and 10% FBS. The cells were maintained at 37°C and a CO₂ concentration of 5% and subcultured at intervals of 2–3 days. Transfection was performed once the cells in a six‐well plate reached 60%–85% confluence.

### 2.5 Minigene Assay In Vitro

The exon capture vector for the minigene assay was pSPL3, with two restriction enzyme sites: XhoI and NheI. In addition, PCR amplification was performed on exon 12 of *COL4A3* along with its flanking introns 11 and 12 with the following set of primers: forward 5′‐accagaattctggagctcgagGTAGACTACAGTTCATATGATGTAACAG‐3′ and reverse 5′‐ttgttctcttaatttgctagcCTAAAGACAAAAACACTCAGGAGTGAG‐3′. The wild‐type and mutant plasmids were produced via the ClonExpress II one‐step cloning kit (C112, Vazyme) and the Mut Express II Fast Mutagenesis Kit V2 (C214, Vazyme), respectively. Then, they were transfected into HEK293T cells via the designated transfection medium via Lipofectamine 3000 (Life Technologies). RNA was collected 24 h after transfection and converted into cDNA. The amplification of cDNA was subsequently performed via PCR with primers explicitly designed for pSPL3: SD6‐5′‐TCTGAGTCACCTGGACAACC‐3′ and SA2‐5′‐ATCTCAGTGGTATTTGTGAGC‐3′. The sequence of the PCR product was determined via Sanger sequencing to analyze the splicing structures.

### 2.6 Histological Analysis and Staining

Kidney biopsies were conducted on hospitalized patients and probands diagnosed with TBMN at the Affiliated Hospital of Guizhou Medical University in Guiyang, Guizhou Province, China. The attending pediatric nephrology specialist performed the biopsy on the basis of their clinical judgment. The biopsy material was collected and examined under a stereomicroscope. The samples were then split for tests via light and electron microscopy. The sample that was studied under a light microscope was treated with neutral buffered formalin and then put into a paraffin mixture according to standard methods. After that, the sections were subjected to hematoxylin and eosin (H&E) staining, periodic acid–Schiff (PAS) staining, periodic acid–silver methenamine (PASM) staining, and Masson's trichrome (Masson) staining. The stained sections were photographed via an Olimbas optical microscope.

### 2.7 Transmission Electron Microscopy (TEM)

The electron microscopy laboratory at the Affiliated Hospital of Guizhou Medical University handled and processed the electron microscopy samples. Six electron micrographs were taken from each of the three glomeruli that were randomly selected from the proband.

### 2.8 Confocal and Fluorescence Microscopy

Immunofluorescence staining was conducted on paraffin sections of kidney tissues, and images were acquired via a Nikon A1R Meta confocal microscope. The following antibodies were used: anti‐COL4A3 antibody (dilution 1:50, #7076, Chondrex), anti‐COL4A4 antibody (dilution 1:50, #7073, Chondrex), anti‐COL4A5 antibody (dilution 1:50, ab231957, Abcam), goat polyclonal secondary antibody to rat IgG H&L Alexa Fluor 647 (dilution 1:400, ab150159, Abcam), goat polyclonal secondary antibody to rabbit IgG H&L Alexa Fluor 488 (dilution 1:400, ab150077, Abcam), and DAPI (dilution 1:1000, C1002, Beyotime).

### 2.9 Statistical Analysis

To organize the metric data that followed a normal distribution, the clinical data were evaluated via descriptive statistical approaches, such as computing the mean and standard deviation.

## 3 Results

### 3.1 The Clinical Phenotypes of the Proband

**FIGURE 1:**
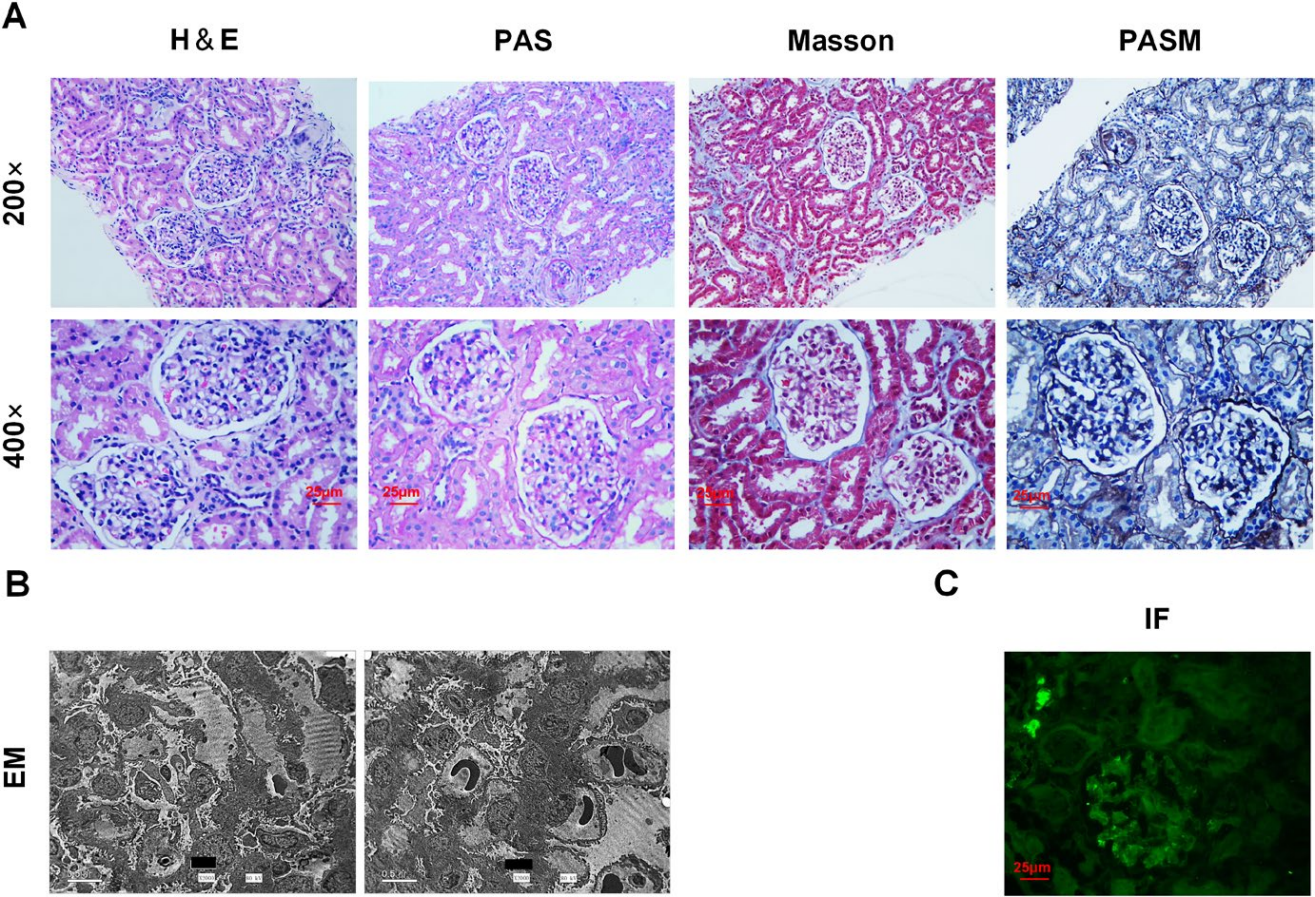
The proband's kidney biopsies revealed GBM thinning. (A) Light microscopy revealed segmental sclerosis of a glomerulus (1SS), a mild segmental proliferation of mesangial cells and the matrix, and segmental vacuolar degeneration of the GBM. (B) Electron microscopy image showing conspicuous thinning of the glomerular basement membrane, segmental wrinkling of the GBM, and segmental fusion of the podocyte foot processes. (C) IF staining: Suspected IgM deposition in the kidney tissue of the proband.

The participant in this study was an 8‐year‐old female who developed the disease at an early stage of 4 years, with recurring microscopic hematuria and microalbuminuria without any discernible etiology. Comprehensive evaluations of hepatic and renal function, as well as serum concentrations of antibodies associated with autoimmune disorders, revealed that the results were within normal limits for this patient. Detailed findings from the serum renal function tests and urinalysis are provided in Tables 1 and 2, respectively. The proband did not have any hearing or vision problems. After 3 years of irregular administration of captopril, the response was not good, so a kidney biopsy was performed. The proband's kidney pathology included H&E, PAS, PASM, and Masson staining. The kidney biopsy revealed suspicious IgM deposits, but no additional immunological deposits were observed (Figure 1C). There were 36 glomeruli, including 1 with glomerular segmental sclerosis with no crescents, mild segmental hyperplasia of mesangial cells and the mesangial matrix, segmental vacuolar degeneration of the glomerulus basement membrane (GBM), no significant photophilic deposition, focal atrophy of renal tubules, vacuolar and granular degeneration of epithelial cells, intracavitary protein tubule formation, and focal lymphoid and monocyte infiltration of the renal interstitium (Figure 1A). Electron microscopy revealed that the mesangial cells and stroma were slightly hyperplastic, the foot processes were segmentally fused, the GBM was thin, and there were no electron‐dense deposits (Figure 1B). The pathologic features of the proband revealed that thin basement membrane nephropathy was more likely (Figure 1A,B). Through an investigation of the family history, it was ascertained that the proband's parents were first cousins. The father presented with isolated microscopic hematuria, maintained normal serum renal function, and exhibited no ocular or auditory involvement.

**TABLE 1:** Urine analysis in proband.

Characteristic	Result	Reference range
Urinary albumin	276.27 ± 70.94↑	< 150 mg/L
Total 24‐h urinary protein	152.50 ± 27.57↑	< 141 mg/24 h
Urinary microalbumin	20.80 ± 19.24↑	0–1.9 mg/L
Urinary erythrocytes	3586.58 ± 2316.81↑	0–4/uL

*Note:* The black arrows “↑” represent the increase of corresponding detection indicators.

**TABLE 2:** Serum renal function indicators in proband.

Characteristic	Result	Reference range
Urea	4.19	2.2–7.14 mmol/L
Creatinine	44.00	14–60 μmol/L
Uric acid	301.00	100–410 μmol/L
Cystatin C	1.14↑	0.59–1.03 mg/L
Corrected Ccr	155.79	—

*Note:* The black arrows “↑” represent the increase of corresponding detection indicators. Ccr, endogenous creatinine clearance rate.

**FIGURE 2:**
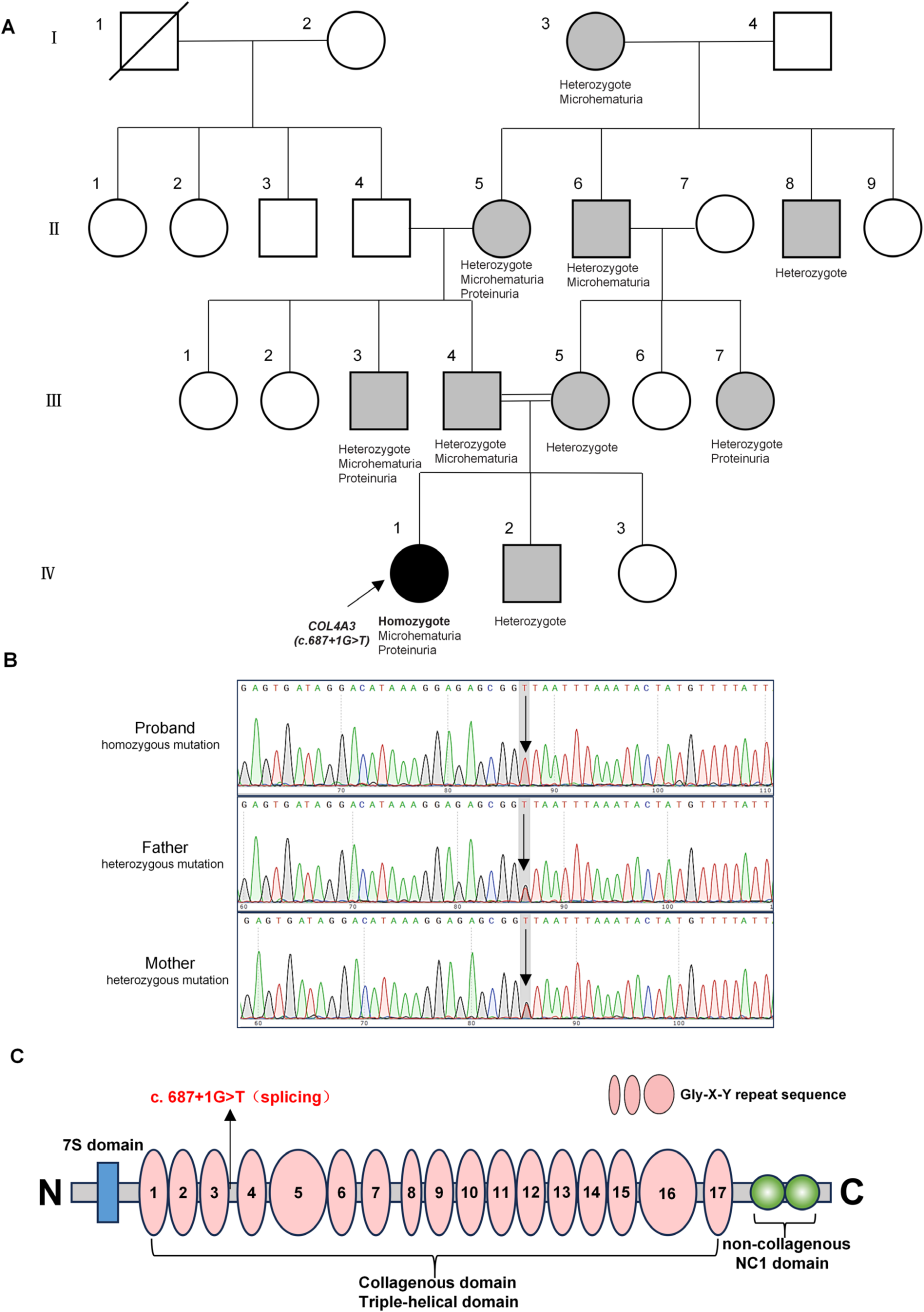
Identification of the mutation *(c.687 + 1G > T)* in *COL4A3* in the family. (A) Pedigree of the proband's family across four generations. (B) Sanger sequencing results of the proband and her parents. (C) Schematic representation of the type IV collagen alpha 3 chain protein domains. The gray circles or boxes represent individuals with heterozygous mutations in *COL4A3 (c.687 + 1G > T)*. The black circle represents the proband with a homozygous mutation in *COL4A3 (c.687 + 1G > T)*. Two parallel horizontal lines indicate consanguinity. The black vertical arrows represent the new mutation.

In contrast, the mother, as well as the younger brother and sister, displayed no clinical phenotypes indicative of AS. To further elucidate the genetic mechanisms underlying the disease, we performed next‐generation sequencing (NGS) analysis on peripheral blood samples from the proband and their family members (Figure 2A). This analysis identified one individual with a homozygous mutation, who is the proband in this family, and nine carriers of heterozygous mutations. Among these nine heterozygous carriers, five exhibited microscopic hematuria, with or without proteinuria; one exhibited microalbuminuria; and three had normal urinalysis results. None of the family members demonstrated any signs of renal function impairment, hearing loss, or visual disturbances.

### 3.2 Identification of a Homozygous Mutation (*c.687 + 1G > T*) in *COL4A3*

As depicted in the family pedigree, we discovered a homozygous variant in *COL4A3* on chromosome 2 of this proband (IV‐1), specifically *c.687 + 1G > T* (NM_000091.5), which represents a distinctive splicing mutation (Table S2 online). The mutation is found in the intron's splicing region. The variant is absent in population databases (ExAC no frequency). The ClinVar database (https://www.ncbi.nlm.nih.gov/clinvar/) lists this site as likely pathogenic (Variant ID: 1067774), but this variant has not been detected in individuals with *COL4A3*‐related diseases. The variant was subsequently verified via Sanger sequencing. The variant was also confirmed to be inherited from her parents (III‐4, III‐5), who both carried a heterozygous variant (Figure 2B). In addition, domain analysis revealed that this mutation site may affect the collagenous domain (Figure 2C).

### 3.3 The In Silico Prediction of the *COL4A3* (*c.687 +* *1G* *> T*) Mutation Indicates That This Mutation Likely Has Detrimental Effects

**FIGURE 3:**
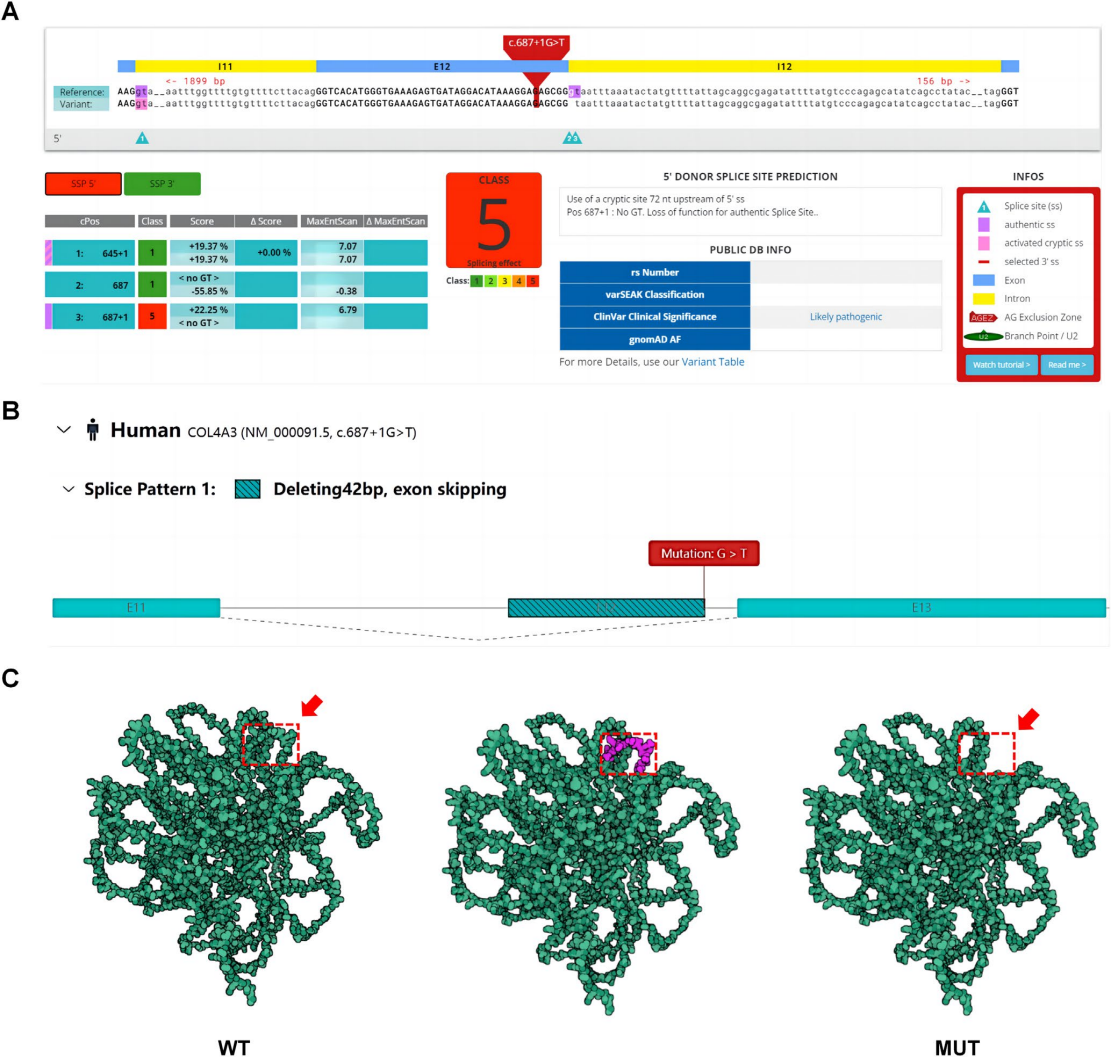
*COL4A3 (c.687 + 1G > T)* mutation according to bioinformatics prediction. (A) This mutation site is predicted to affect splicing. Predicted by varSEAK. (B) This mutation site is predicted to cause abnormal splicing, resulting in exon skipping. (C) Protein crystal structure of the type IV collagen alpha 3 chain. The mutation leads to the deletion of some aa, as indicated by the red dashed box and red arrows. MUT, mutant type; WT, wild type.

We utilized a variety of instruments, including the American College of Medical Genetics (ACMG), Human Splicing Finder (HSF), MutationTaster, SpliceAI, and varSEAK online, to predict the pathogenicity of mutations. The tools mentioned above revealed that the *c.687 + 1G > T* mutation was likely pathogenic (PVS1+ PM2_Supporting) and was predicted to cause abnormal splicing (Figure 3A and Table S3 online). In addition, when the Rare Disease Data Center‐RNA Splicer (RDDC‐RNA Splicer) online tool is used, this site mutation is expected to cause exon 12 to skip (Figure 3B). Moreover, the 3D structure of this protein, which might lack some aa, was predicted via the AlphaFold protein structure database (Figure 3C).

### 3.4 The Minigene Assay Confirmed That *c.687 +* *1G* *> T* in *COL4A3* Causes Exon Skipping

**FIGURE 4:**
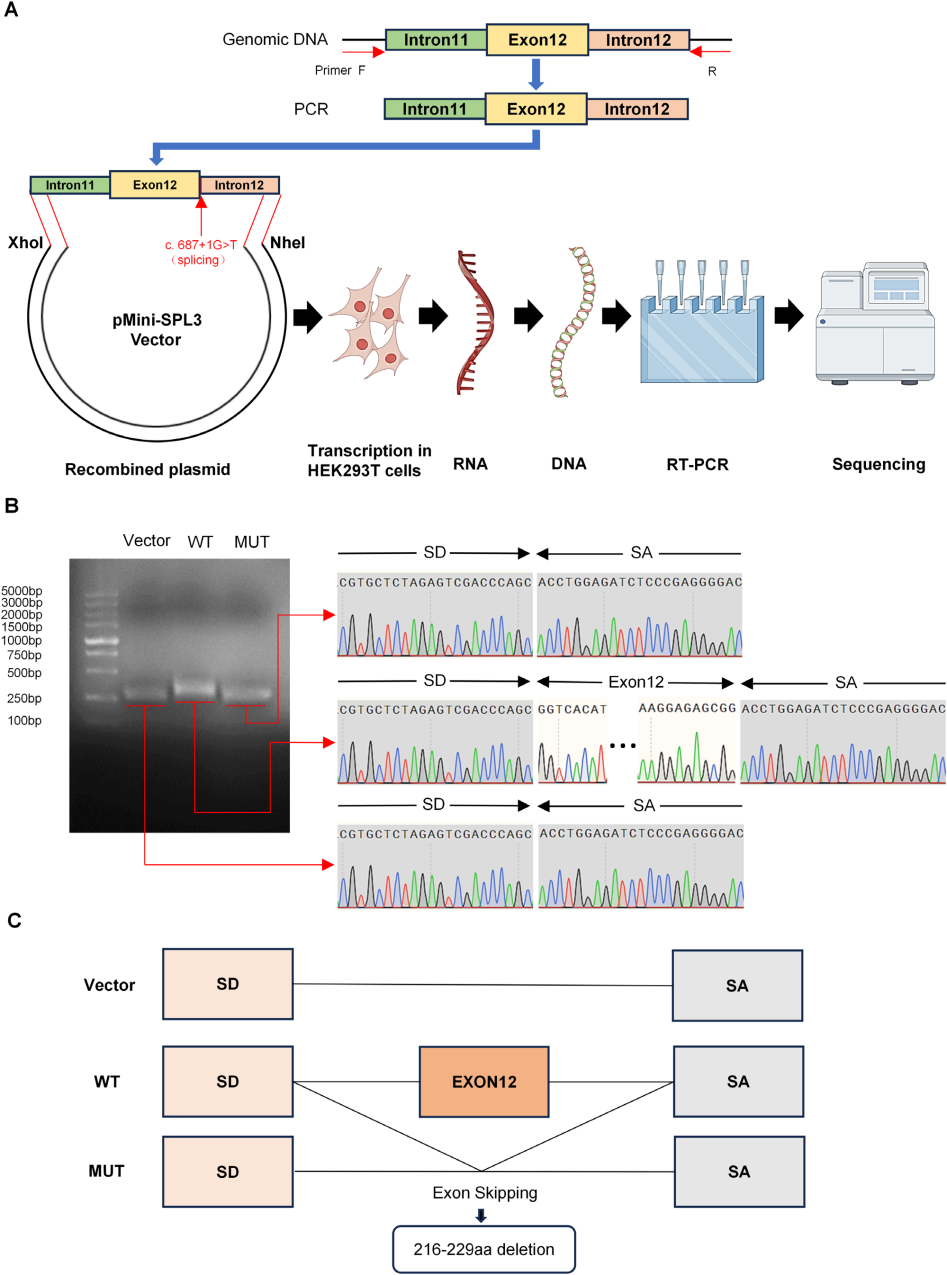
In vitro minigene assay confirming mutation‐induced abnormal splicing. (A) Workflow of the minigene experiment. Generated by Figdraw. (B) RNA from transfected HEK293T cells was subjected to gel electrophoresis to analyze the RT–PCR products. (C) The sequencing findings presented above demonstrate that the splicing variant *(c.687 + 1G > T)* results in the exclusion of exon 12 and the removal of aa 216 to 229. MUT, mutant type; WT, wild type; Vector, empty vector.

After confirming that the minigene test is a dependable method for assessing possible splicing variants functionally, we created wild‐type (WT) and mutant (MUT) plasmids that specifically target the *c.687 + 1G > T* mutation, which we then transfected into HEK 293 T cells. Then, reverse transcription was performed on the isolated RNA, and PCR was used to amplify the cDNA (Figure 4A). The results demonstrated that the WT plasmid's transcriptional sequences were as predicted and included the whole mRNA product produced by exon 12. Sequencing of the mutant plasmid revealed that exon 12 skipping and mRNA splicing were impacted by the *c.687 + 1G > T* mutation (Figure 4B). It is anticipated that this mutation will lead to a loss of 14 aa, along with a partial loss of the collagenous portion of the type IV collagen α3 chain protein, which could impact the function of the protein (Figure 4C).

### 3.5 *COL4A3* Mutation Affects the Expression of the Target Protein and Type IV Collagen α3α4α5 Heterotrimers

**FIGURE 5:**
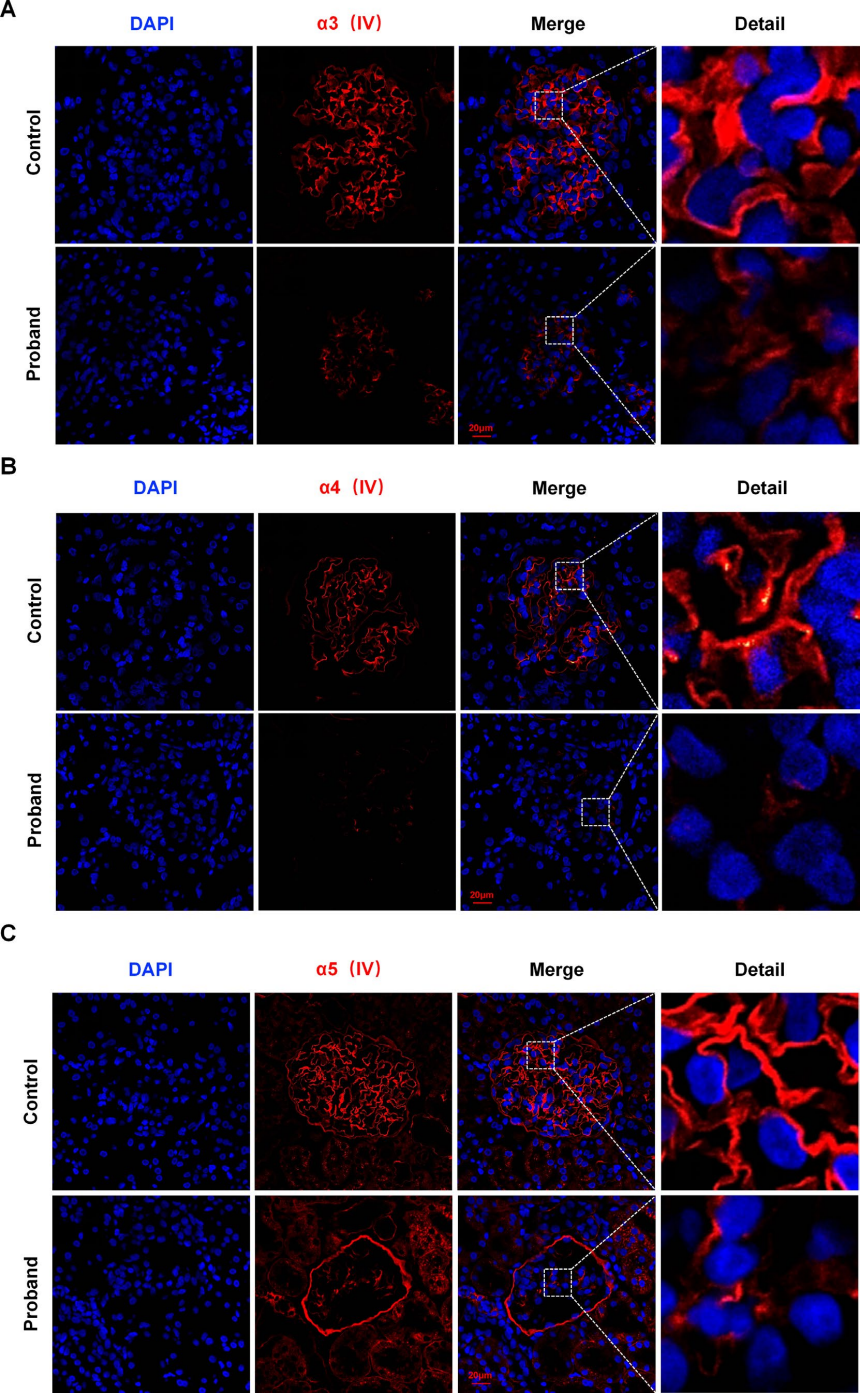
IF staining images of the proband's renal tissue paraffin sections. (A) Compared with that in the control group, the expression of the α3 chain of type IV collagen in the GBM significantly decreased. (B,C) The expression of type IV collagen α4 and α5 chains in the GBM is significantly reduced, resulting in noticeable partial loss.

To determine how this mutation affects the production of type IV collagen, we stained paraffin slices of kidney tissue from the proband and the control group with an immunofluorescence (IF) dye. In the proband, a substantial reduction in the production of the collagen IV alpha3 chain was observed (Figure 5A). Furthermore, in comparison with the control group, there was a partial absence and a marked decrease in the expression of the type IV collagen alpha4 and alpha5 chains (Figure 5B,C).

## 4 Discussion

This study provides the molecular profile of a patient with proteinuria and hematuria but no renal failure, hearing loss, or abnormalities of the eyes. A thin glomerular basement membrane was observed during kidney biopsy, yet Flinter's criteria for diagnosing AS were not met (Flinter et al. 1988). However, not all afflicted individuals with AS show typical abnormalities via electron microscopy, and in some patients (10%–20%), especially children, the only change observed is that the glomerular basement membrane becomes thinner throughout (Hanson et al. 2011; Heidet and Gubler 2009). Therefore, the next‐generation sequencing results revealed a homozygous variant in this patient, *COL4A3 (c. 687 + 1G > T)*, and ACMG prediction indicated that it was a likely pathogenic variant. The causal relationship between the mutation and the disease in the proband was ultimately determined by integrating in vitro studies investigating the splicing impact of *COL4A3* transcripts. Consequently, she was diagnosed with AS. Both of her parents, who are consanguineous, carry a heterozygous *COL4A3* mutation *(c. 687 + 1G > T)*. Through validation with next‐generation sequencing, nine carriers of this mutation were identified among the family members, among whom five presented with microscopic hematuria, with or without proteinuria; one had microalbuminuria; and three had normal urinalysis results. These observations suggest that the mutation follows an autosomal recessive inheritance pattern. ARAS is linked to *COL4A3* or *COL4A4* variants that are either homozygous or compound heterozygous. Hearing loss and blindness are more common in individuals who are homozygous or compound heterozygous for ARAS, and they move quickly to ESKD (Heidet and Gubler 2009). The prevalence of microscopic hematuria, proteinuria, chronic renal failure (CRF), loss of hearing, anterior lenticonus, and dot‐and‐fleck retinopathy is considerably greater in biallelic individuals than in heterozygous patients (Nabais Sa et al. 2015). In addition, *COL4A3*‐related individuals carrying heterozygous mutations have a vast spectrum of phenotypes, with some showing no symptoms at all and others showing intermittent or permanent microhematuria (Heidet et al. 2001). The results of this study are consistent with those of the reports mentioned above.

With the rapid advancement of high‐throughput sequencing technology, an increasing number of splicing abnormalities have been reported in AS. Jais et al. reported a 70% likelihood of developing ESKD by thirty birthdays in individuals with splicing mutations (Jais et al. 2000). Furthermore, the mean number of years of ESKD individuals with splicing changes has been reported to be 28 years (Bekheirnia et al. 2010). Thus, AS patients with splicing mutations have a greater probability of early progression to ESKD. Table S4 provides a comprehensive summary of *COL4A3* splicing mutations reported in prior studies in patients with varying ages of onset and most patients with renal phenotypes. Among these patients, homozygous or compound heterozygous mutations accounted for 43/86 (50%), and heterozygous mutations accounted for 43/86 (50%). Furthermore, among individuals with homozygous or compound heterozygous mutations, 22/43 (51.2%) experienced visual lesions or hearing loss, and 14/43 (32.6%) acquired ESKD. In this study, the proband harbored homozygous splicing mutations and presented with mild hematuria and proteinuria, but no renal dysfunction or extrarenal manifestations were observed, which may be related to her relatively young age. Nevertheless, Story Helen et al. were the first to describe a mild kidney phenotype in ARAS, with some adult and child patients still having normal kidney function (Storey et al. 2013). Therefore, follow‐up with this proband and regular monitoring of renal function and extrarenal manifestations are still necessary.

However, further research has revealed that heterozygous mutations in *COL4A3* can result in a spectrum of symptoms, including ocular and auditory issues, progressive kidney disease leading to ESKD, and asymptomatic cases, even within the same family (Gross et al. 2003; Heidet et al. 2001). In this study, among 22 family members, nine were identified as carriers of heterozygous mutations; five exhibited microscopic hematuria, with or without proteinuria; one had microalbuminuria, and three had normal urinalysis results. Nonetheless, none of these carriers presented with renal function impairment, hearing loss, or vision problems. Regular monitoring is recommended for these family members, and special attention should be given to subsequent follow‐ups for the emergence of hypertension, increased urinary albumin and red blood cell counts, renal failure, and damage to the eyes and ears.

The *COL4A3* gene encodes the 1670 aa that makes up the a3 chain of collagen IV, which is found in the 2q36‐37 region of the genome and consists of 52 exons and 51 introns (Storey et al. 2013; Xie et al. 2014). The development of a triple helix structure comprising type IV collagen α3, α4, and α5 chains plays a crucial role in the integrity and functionality of the GBM (Kashtan et al. 2018). Collagen IV chains are large proteins comprising a short 7S domain at the beginning, an extended collagen region with approximately 1400 Gly‐X‐Y repeats broken up by 20 small NC domains, and a short, 230 amino acid NC domain at the end (Naylor, Morais, and Lennon 2021). The proband's *COL4A3* mutation was located in the collagen domain in this study. The analysis of the minigene revealed an anomalous splicing pattern, leading to the deletion of 14 aa within the Gly‐X‐Y repetition portion of the long central trihelix domain of the α3 (IV) chain.

The splice‐site mutation observed in this AS family is predicted to cause a deletion of 14 amino acids within the α3 (IV) chain. This alteration results in the production of a truncated collagen chain, eliminating four Gly‐X‐Y sequences (Mariyama et al. 1994), which aligns with previous reports of deletions within the collagen domain framework (van der Loop et al. 2000). Notably, this in‐frame deletion does not lead to premature termination of protein synthesis, nor does it disrupt the reading frame. Consequently, the renal function of the patient and the affected family members is presently maintained, with no extrarenal symptoms observed at this time. However, the possibility of the emergence of such symptoms later in life cannot be excluded.

The aggregation of the α3 (IV) chain in the endoplasmic reticulum (ER) and its subsequent formation of helical heterotrimers with the α4(IV) and α5(IV) chains have been previously documented. These heterotrimers are secreted into the GBM, establishing an intricate network comprising multiple structural constituents (Pieri et al. 2014). Suppose that a pathogenic coding gene mutation has caused a malfunction in any of those chains. The disintegration of trimers in the highly organized GBM, which is usually caused by the suppression of the expression of the other two chains, may subsequently result in renal failure (Heidet et al. 2000; Liang et al. 2023; Massella et al. 2013). Furthermore, previous studies have indicated that *COL4A3* knockout mice lack type IV collagen a4 and a5 chains in the glomeruli (Cosgrove et al. 1996; Miner and Sanes 1996). In our investigation, immunofluorescence staining of kidney paraffin sections from the proband revealed a decrease in the expression and partial absence of type IV collagen α3, a4, and a5 chains in the GBM sample compared with those in the control sample. Thus, this mutation induces alterations in the α3 chain of type IV collagen, potentially leading to impairment of the trimeric network formed by type IV collagen a3a4a5 through integration of the modified α3 chain within the trimeric structure. It elicits structural anomalies in the GBM, disrupting the integrity of the glomerular filtration barrier(GFB) and resulting in the extravasation of erythrocytes and albumin. However, the underlying molecular mechanism remains unknown and warrants further investigation.

Regrettably, a definitive cure for AS has not yet been identified. Angiotensin‐converting enzyme inhibitors (ACEIs) and angiotensin II receptor blockers (ARBs) have been shown in numerous studies to be effective therapeutic approaches for patients with AS, significantly postponing the onset of renal failure in these individuals (Boeckhaus et al. 2022; Nozu et al. 2019; Yamamura et al. 2020; Zeng et al. 2023; Zhang et al. 2021). In the present study, the proband received oral administration of ACEIs, resulting in a notable decrease in urinary protein and hematuria relative to her prior treatment regimen, indicating the effectiveness of the therapy. Currently, her health status is stable, with normal renal function and an absence of extrarenal symptoms.

## 5 Conclusions

To summarize, this study investigated a familial example of ARAS and clarified its correlation with *COL4A3* splicing mutations. This mutation results in abnormal splicing of *COL4A3*, which in turn causes abnormal translation. This abnormal translation prevents the assembly of the α3α4α5 chain of collagen IV, which affects the shape and function of the GBM. This study enriches the genetic map of ARAS and provides a reference for clinical genetic counseling and further prenatal diagnosis. Moreover, because *COL4A3 (c.687 + 1G > T)* is now recognized as a likely pathogenic variant in the ClinVar database, our study is the first to report this variant in a human disease and strongly supports this causative effect experimentally.

## Author Contributions

X.Y. and T.Z. created and planned the research. D.C. conducted the experiments and wrote the manuscript. J.R., Y.Z., S.H., L.D., C.Y., and Q.B. acquired and analyzed clinical data. X.Y. and L.Z. provided critical intellectual input for essential content during the manuscript revision. All authors had full permission to review the data, evaluate draft manuscripts, and approve the final version.

## Ethics Statement

The study followed the principles of the Declaration of Helsinki and the procedure was approved by the Medical Ethics Committee of Affiliated Hospital of Guizhou Medical University(No.2024030 K).

## Consent

Written informed consent was obtained from the parents of the patients for publication of the information contained within this article.

## Conflicts of Interest

The authors declare no conflicts of interest.

## Supporting information


Tables S1–S3.

Table S4.

## Data Availability

The complete dataset from this study can be obtained by contacting the corresponding author upon request.
