# Supplementary material for: Effects of a Novel COL4A3 Homozygous/Heterozygous Splicing Mutation on the Mild Phenotype in a Family With Autosomal Recessive Alport Syndrome and a Literature Review
Source: Mol Genet Genomic Med. 2025 Feb 9;13(2):e70053. doi: 10.1002/mgg3.70053 (PMC11807844; doi:10.1002/mgg3.70053)
Supplement: Supplementary file 1 — Tables S1–S3. [file MGG3-13-e70053-s001.pdf]

Effects of a novel *COL4A3* homozygous/heterozygous splicing mutation on the mild phenotype in a family with autosomal recessive Alport syndrome and a literature review

Dan Chen[1], Li Zhang[1],Jing Rao[1], Yan Zhou[1], Lujun Dai[2],Songsong Huang[2],Chunxia Yang[1], Qiuhan Bian[1], Tao Zhang[1, #],Xiaoyan Yang[1, #]

Author Affiliations:

[1] Department of Pediatrics, Affiliated Hospital of Guizhou Medical University, Guizhou Provincial Children's Medical Center, Guiyang, Guizhou, P.R China.

[2] Department of Pathology, Affiliated Hospital of Guizhou Medical University, Guiyang, Guizhou, P.R China.

Supplementary Table S1.

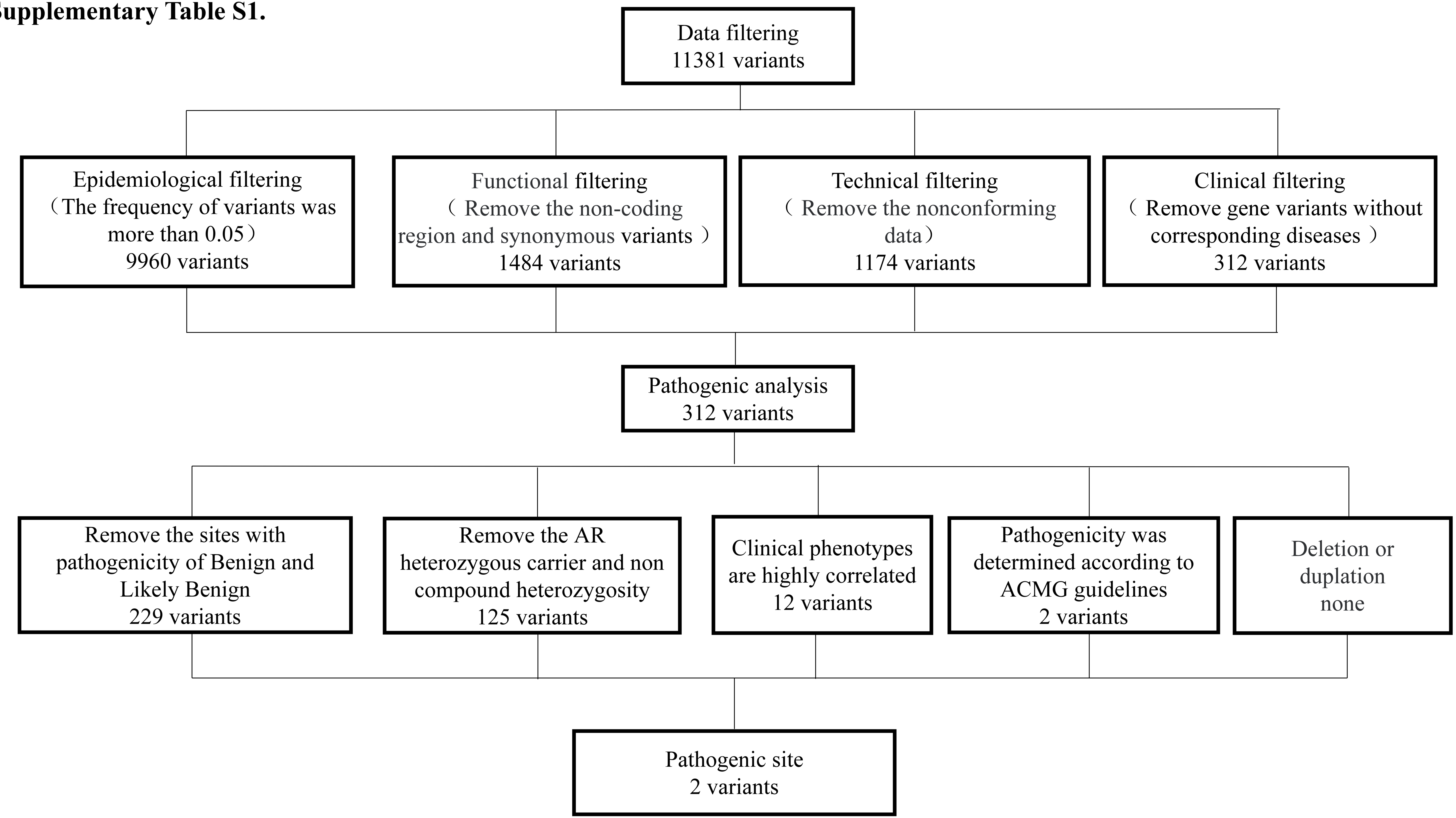

Supplementary Table S2. Single nucleotide polymorphisms in *COL4A3* from the index proband

Gene	Chromosome position	Base （amino acid）	Function	MAF	Prediction	Pathogenic analysis	Inheritance	Type of mutation
COL4A3	chr2: 228118054	c.687+1G>T （Splicing）	Splice variant	-	-	Likely pathogenic	Father、Mother	Germline

Transcript used: RefSeq NM_000091.5. Abbreviations: MAF, minor allele frequency.

Supplementary Table S3.

**ALGORITHM**	***COL4A3 c.687+1G>T*** **PRIDICTION（SCORE）**
**the American College of Medical Genetics(ACMG)**	likely pathogenic（PVS1+PM2_Supporting）
**the Human Splicing Finder (HSF)**	Alteration of the WT Donor site, most probably affecting splicing（84.19>57.05： -32.24%）
**MaxEnt**	Alteration of the WT Donor site, most probably affecting splicing（6.79>-1.72： -125.33%）
**Mutation Taster**	Disease_causing（1）
**SpliceAI**	T（0.91）

Note:Transcript used: RefSeq NM_000091.5; The higher the MutationTaster predicted score, the more likely it was to cause disease; SpliceAI predicted a value greater than 0.2 to demonstrate the influence of splicing.
